# Night-time blood pressure dipping, but not 24-h blood pressure level, is linked to increased 24-h ocular volume slope

**DOI:** 10.5935/0004-2749.2022-0236

**Published:** 2024-07-09

**Authors:** Janusz Skrzypecki, FM Szymański, J. Przybek-Skrzypecka, Justyna Izdebska, A. Ryś-Czaporowska, KJ Filipiak, Jacek P. Szaflik

**Affiliations:** 1 Department of Experimental Physiology and Pathophysiology, Laboratory of Centre for Preclinical Research, Medical University of Warsaw, Warsaw, Poland; 2 Department of Civilization Diseases, Faculty of Medicine, Collegium Medicum, Stefan Cardinal Wyszynski University in Warsaw, Warszawa, Poland; 3 Department of Ophthalmology, SPKSO Ophthalmic Hospital, Medical University of Warsaw, Warsaw, Poland; 4 Institute of Clinical Sciences, Maria Sklodowska-Curie Medical Academy, Warszawa, Poland

**Keywords:** Intraocular pressure, Blood pressure, Contact lens, Glaucoma, Hypertension, Hypotension

## Abstract

**Purpose:**

This study investigated the relationship between blood pressure and intraocular pressure in treatmentnaive, non-glaucoma patients with different blood pressure statuses, focusing on the 24-h ocular volume and nocturnal blood pressure decline.

**Methods:**

Treatment-naive, non-glaucoma patients undergoing hypertension evaluation were enrolled as study participants. Simultaneous 24-h ambulatory blood pressure measurement and 24-h ocular volume recording with a contact lens sensor. We also compared ocular volume curve parameters between normotensive and hypertensive patients, as well as between those with and without nocturnal blood pressure decline.

**Results:**

A total of 21 patients, including 7 normotensive and 14 treatment-naive hypertensive individuals, were included in the study. of them, 11 were dippers and 10 were non-dippers. No significant difference in the 24-h ocular volume slope was observed between the hypertensive and normotensive patients (p=0.284). However, dippers had a significantly higher 24-h ocular volume slope (p=0.004) and nocturnal contact lens sensor output (p=0.041) than non-dippers.

**Conclusion:**

Nocturnal blood pressure decline, rather than the blood pressure level, is associated with the increased 24-h ocular volume slope and nocturnal ocular volume. Further studies are required to determine whether the acceleration of glaucoma progression in dippers is primarily due to low blood pressure, high intraocular pressure, or a combination of both.

## INTRODUCTION

Intraocular pressure (IOP) is the only clinically well-established and modifiable risk factor for glaucoma progression^(^1^)^. However, research indicates that additional attention should be focused on the relationship between blood pressure (BP) levels and IOP in glaucoma patients^(^2^, ^3^)^.

Although BP is known to affect the optic nerve function, the causative link remains unelucidated^(^2^)^. On the one hand, large epidemiological studies have demonstrated that hypertension is a risk factor for glaucoma or that IOP values are positively correlated with systemic BP^(^4^, ^5^)^. By contrast, low systemic BP accelerates glaucoma progression^(^6^, ^7^)^. Notably, several studies have reported that nocturnal over-dipping of systemic BP accelerates the progression of changes in the visual field^(^8^, ^9^)^.

These contradicting observations might be explained by the theory that neurohormonal dysregulation underlying systemic hypotension or hypertension, rather than BP values, increases IOP, thereby damaging the optic nerve^(^10^)^. For instance, at night, physiological values of BP and IOP decrease and increase, respectively^(^11^, ^12^)^. this pattern is hypothesized to reflect the divergent effect of decreased sympathetic activity on BP and IOP^(^10^)^. Experimental studies on cervical gangliectomy have supported this hypothesis. They reported an increase in IOP in the long term ^(^13^)^. IOP lowering was historically based on a non-selective sympathomimetic, that is adrenaline^(^14^)^. Therefore, we hypothesized that low BP, rather than a relative increase in IOP in response to nocturnal sympathetic downregulation, might be the main factor driving the development of glaucomatous changes in some patients with nocturnal hypotension.

Interestingly, to the best of our knowledge, no clinical study has quantitatively correlated an increase in nocturnal IOP and a decrease in BP in non-glaucoma, treatment-naive patients with different BP statuses. This is partly related to the fact that until recently, no method allowed continuous IOP measurements under habitual conditions. SENSIMED Triggerfish (Sensimed AG, Switzerland), a contact lens sensor (CLS) approved by the FDA in 2016, facilitates the recording of changes in the 24-h ocular volume. Interestingly, the peak CLS output correlates in time with peak lOP^(^15^, ^16^)^. Furthermore, although no direct quantitative correlation exists between the ocular volume and IOP, certain parameters, which can be mathematically derived from the ocular volume curve (e.g., the number of large peaks (>90 mVEq), mean peak ratio, and wake-to-sleep slope), were found to be viable surrogate parameters for IOP in terms of glaucoma progression (for a more detailed description about ocular volume changes, please refer to the original article by Moraes et al.)^(^17^)^.

We here compared 24-h BP and changes in 24-h ocular volume in normotensive and treatment-naive hypertensive, non-glaucoma patients as well as in patients with and without nocturnal BP decline, that is, in groups with different sympathetic activities.

## METHODS

This study was approved by the Medical University of Warsaw’s Bioethical Committee. 1t involved patients who were undergoing systemic hypertension evaluation at the Department of Cardiology. The patients signed a written consent after they were informed about the study protocol.

Eligible patients were adults, had no history of glaucoma or ocular hypertension, and were not using any medications to lower intraocular or systemic hypertension. Patients with any secondary form of hypertension (e.g., renal stenosis, Cushing’s syndrome, or pheochromocytoma) were excluded. Furthermore, patients with any abnormality observed during the eye examination that may increase IOP (e.g., an angle of grade 1 or 0 in the Schaffer classification and a previous ocular surgery) were excluded from the study. Additionally, patients with any abnormality of the cornea or eye surface that prevented contact lens fitting were excluded.

Simultaneous 24-h ambulatory blood pressure monitoring (ABPM) (Schiller BR 102 plus, Switzerland) was performed on the included participants to identify 7 normotensive and 14 treatment-naive, hypertensive patients. 1n all included patients, 24-h ocular volume change was simultaneously monitored using the SENS1MED Triggerfish system, in which a strain gauge embedded in a soft contact lens is used for measuring dimensional changes in the limbal area, correlating with the ocular volume and IOP. Day and night data were extracted using the blink recordings of the CLS.

The left or right eye was selected for the study at the patient’s discretion. Routine eye examination, which involved slit-lamp evaluation of anterior and posterior segments, Goldmann applanation tonometry, gonioscopy, retinal nerve fiber layer (RNFL) thickness measurements (Triton OCT, Topcon, Japan), keratometry (autore-fractor keratometer GR-3100K, Grand Seiko, Japan), and 24-2 visual field examination (Humphrey Field Analyzer 11, Zeiss, Germany), was conducted at the Department of Ophthalmology.

The BP Holter devices and CLS were fitted between 9 am and 11 am. BP was measured every 20 min, both while awake and asleep, with dedicated software automatically generating BP curves and mean measurements. The CLS recorded ocular volume-related parameters every 5 min over 24 h. The devices were removed after 24-h. BP Holter and CLS recordings were eligible for further analysis until uninterrupted 24-h recordings were available.

For this study, each participant was assigned two labels in accordance with cardiology guidelines, one from each category: (A) hypertensive patients 24-h SBP and/or 24-h DBP >130 mmHg and 80 mmHg, or normotensive patients (B); nocturnal dippers with a 10%-20% nocturnal BP drop or nocturnal non-dippers without nocturnal BP drop^(^18^)^.

### Statistical analysis

We also compared a group of previously analyzed Triggerfish parameters, that is, the slope of the regression line of the 24-h ocular volume curve, mean day-time and night-time CLS output, and variability of the mean during the day and the night.

### The slope of the regression line of the 24-h ocular volume curve

This parameter was modeled from the single-point measurements conducted over 24-h.

### Mean day-time and night-time CLS output

Mean day-time and night-time output was calculated from the single-point measurements over day and night, respectively. Based on the CLS output, the night was defined as a period without blinking.

### Variability of the mean

This parameter was calculated from the single-point measurements conducted over 24-h.

### Number of peaks above 90 mVEq

Only large peaks were selected for the analysis so as to avoid including artifacts. These parameters have been used by other authors to validate CLS measurements^(^17^, ^19^)^.

The Kolmogorov-Smirnov test was performed to test for normal distribution. Student’s t-test, Mann-Whitney U test, regression analysis, and the chi-square test for contingency tables were used to calculate statistical significance. Bonferroni correction was applied for multiple comparisons. The slope of the regression line was calculated using the Prism (Graphpad, USA) algorithm, and both positive and negative areas were included.

Correlation analysis was performed to evaluate the relationship between BP, lOP, ocular volume, and RNFL. Pearson correlation coefficient (values from -1 to +1) was employed to mathematically describe the correlation. +1 describes full linear correlation, 0 indicates no correlation, and -1 indicates full inverse correlation.

The statistical significance level was set at p<0.05.

## RESULTS

This study included 24 Caucasian patients who underwent simultaneous ABPM and 24-h ocular volume recording. Three patients were excluded from the analysis because their 24-h ocular volume recordings were incomplete. Then, 7 normotensive and 14 treatment-naive hypertensive patients were remaining. Table 1 presents the demographic parameters of the included patients. Among these patients, 11 were categorized as dippers and 10 as non-dippers (Table 1). No significant differences in 24-h systolic BP (SBP) (p = 0.858) or 24-h diastolic BP (DBP) (p = 0.695) were observed between the dippers and non-dippers (Table 2). However, hypertensive patients had significantly higher 24-h SBP (p=0.0006) and 24-h DBP (p=0.028) than their normotensive counterparts (Table 2). Additionally, no significant differences in baseline lOP, flat and steep keratometry, or RNFL thickness were observed between the dippers and non-dippers, as well as between the normotensive and hypertensive patients (Table 1).

**Table 1:** Characteristics of the group

	All	Normotensive	Hypertensive	p1	Dippers	Non-dippers	p2
Male	13	4	9	0.999	6	7	0.659
Female	8	3	5	0.999	5	3	0.659
Age (years)	50 ± 2.6	53.6 ± 2.6	48.9 ± 3.5	0.117	46.8 ± 3.4	54 ± 3.7	0.328
K flat (D)	43.2 ± 0.3	44.1 ± 0.5	42.8 ± 0.4	0.196	42.4 ± 0.6	42.9 ± 0.3	0.356
K steep (D)	44.1 ± 0.4	45.3 ± 0.5	43.6 ± 0.4	0.190	44.3 ± 0.7	43.8 ± 0.4	0.401
Baseline lOP (mmHg)	11.1 ± 0.5	11.7 ± 1.3	10.8 ± 0.4	0.332	10.5 ± 0.8	11.6 ± 0.7	0.461
lOP after 24-h (mmHg)	11.9 ± 0.6	11.6 ± 1.3	12 ± 0.6	0.771	11.2 ± 1.0	13 ± 0.8	0.239
Mean RNFL thickness	97.9 ± 1.4	96.7 ± 3.5	98.8 ± 0.7	0.721	97.3 ± 1.8	98.4 ± 2.2	0.956

All results are expressed as the mean ± standard error of the mean (SEM).

K= keratometry; IOP= intraocular pressure; RNFL= retinal nerve fiber layer.

p1: normotensive vs. hypertensive; p2: dippers vs. non-dippers; p<0.05 level of statistical significance.

**Table 2:** Results of 24-h ambulatory blood pressure measurement

	All	Normotensive	Hypertensive	pi	Dippers	Non-dippers	p2
24-h SBP (mmHg)	138 ± 4	119 ± 4	139 ± 4	0.0006	137 ± 7	139 ± 5	0.858
24-h DBP (mmHg)	84 ± 3	76 ± 2	88 ± 4	0.028	83 ± 5	85 ± 3	0.695
Day SBP (mmHg)	139 ± 4	124 ± 4	148 ± 5	0.004	142 ± 7	136 ± 5	0.267
Day DBP (mmHg)	86 ± 3	80 ± 3	91 ± 4	0.162	87 ± 5.3	84 ± 4	0.364
Night SBP (mmHg)	125 ± 4	109 ± 4	135 ± 4	0.005	122 ± 7	131 ± 5	0.644
Night DBP (mmHg)	75 ± 3	68 ± 2	79 ± 4	0.08	73 ± 5	76 ± 3	0.795
24-h HR (no/min)	73 ± 2	73 ± 3	72 ± 2	0.681	72 ± 3	75 ± 2	0.394

All results are expressed as the mean ±standard error of the mean (SEM)

p1: normotensive vs. hypertensive; p2: dippers vs. non-dippers; p<0.05 level of statistical significance; SBP - systolic blood pressure, DBP - iastolic blood pressure, 24-h HR - average heart rate over 24 h.

**Figure 1:**
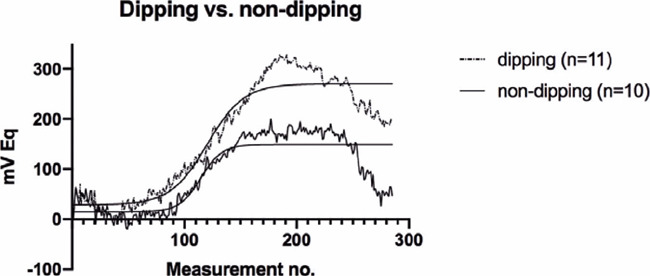
The 24-h ocular volume curve and 24-h ocular volume slope in patients without and with night-time blood pressure (BP) dipping.

**Figure 2:**
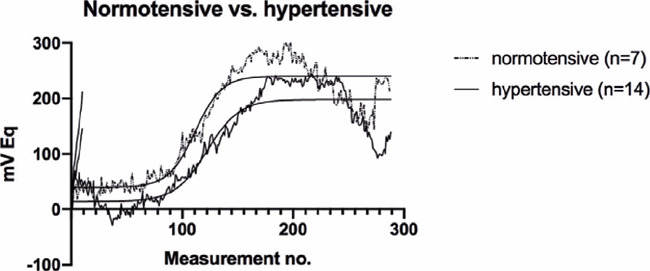
The 24-h ocular volume curve and 24-h ocular volume slope in patients without and with systemic hypertension.

Upon further analysis, we noted that dippers had a significantly higher 24-h ocular volume slope (p=0.004) and mean nocturnal CLS output (p=0.041) than non-dippers (Figure 1, Table 3). By contrast, no significant differences in these parameters were observed between the normotensive and hypertensive patients (p=0.285 and p=0.991, respectively) (Figure 2, Table 3). Moreover, no significant differences in mean day-time CLS output, variability of the mean, or the number of peaks over 90 mVEq were detected between the non-dippers and dippers, or between the normotensive and hypertensive patients (Table 3).

Dipping - patients with night-time BP dipping; non-dipping - patients without night-time BP dipping.

P=0.004 dipping vs. non-dipping 24-h ocular volume slope.

**Table 3:** Results of 24-h ocular volume measurement

	All	Normotensive	Hypertensive	P1	Dippers	Non-dippers	P2
Mean CLS output-day(mVEq)	73 ± 23	92 ± 51	64 ±24	0.998	109 ± 43	34 ± 25	0.113
Mean CLS output-night(mVEq)	253 ± 33	280 ± 68	239 ± 35	0.991	315 ± 54	185 ± 37	0.041
Variability of the mean (day)	54 ± 4	49 ± 9	56 ± 6	0.352	64 ± 9	45 ± 4	0.092
Variability of the mean (night)	40 ± 7	49 ± 19	35 ± 3	0.288	44 ± 16	36 ± 4	0.598
Number of large peaks (>90 mV), day	3.6 ± 0.7	2.7 ± 1.2	4.1 ± 0.9	0.248	3.8 ± 1.2	3.4 ± 1	0.787
Number of large peaks (>90 mV), night	0.4 ± 0.2	0.4 ± 0.3	0.4 ± 0.2	0.975	0.4 ± 0.3	0.4 ± 0.2	0.905

All results are expressed as the mean ±standard error of the mean (SEM)

p1: normotensive vs. hypertensive; p2: dippers vs. non-dippers; p<0.05 level of statistical significance, CLS - contact lens sensor.

The correlation analysis (Table 4) revealed no significant relationships between 24-h SBP, 24-h DBP, day-time SBP, night-time SBP, day-time DBP, night-time DBP, and lOP, as well as 24-h, day-time, and night-time area under the curve (AUC) of the ocular volume. Furthermore, RNFL thickness and 24-h AUC of the ocular volume exhibited no significant correlation (p=0.852).

**Table 4:** Correlation analysis between blood pressure parameters and baseline intraocular pressure as well as ocular volume parameters

		IOP	24-h AUC ocular volume	Day-time AUC ocular volume	Night-time AUC ocular volume
24-h SBP	r	0.063	−0.107	−0.041	−0.091
	^p^	0.79	0.64	0.86	0.69
24-h DBP	r	−0.049	0.018	0.024	0.023
	^p^	0.83	0.938	0.915	0.920
Day-time SBP	r	0.035	0.185	0.272	0.247
	^p^	0.879	0.421	0.233	0.280
Day-time DBP	r	−0.041	0.167	0.171	0.191
	^p^	0.859	0.469	0.459	0.407
Night-time SBP	r	0.148	0.035	0.304	0.160
	^p^	0.522	0.879	0.181	0.488
Night-time DBP	r	0.052	0.074	0.194	0.138
	^p^	0.822	0.750	0.399	0.549

SBP= systolic blood pressure; DBP= diastolic blood pressure; AUC= area under the curve; r= Pearson’s correlation coefficient; p<0.05 - level of statistical significance.

In summary, dippers exhibited a significantly higher 24-h ocular volume slope and mean night-time CLS output than non-dippers. However, no significant differences in these parameters were observed between the normotensive and hypertensive patients. Additionally, significant correlations were not observed between various BP parameters and IOP, as well as between RNFL thickness and 24-h AUC of the ocular volume.

## DISCUSSION

We here observed a significant difference in the 24-h ocular volume slope and the nocturnal mean of the CLS output between the patients with and without nocturnal BP decline, but not between the normotensive and treatment-naive, hypertensive patients.

IOP is currently the only well-established and modifiable risk factor for glaucoma development and progression^(^20^)^. However, because up to 40% of normal IOP patients develop glaucoma, the BP status is an additional risk factor for the disease^(^2^)^. Existing data are somewhat contradictory, with large epidemiological studies exhibiting a positive correlation between BP and IOP, whereas other studies indicate a correlation between nocturnal BP decline and disease progression^(^21^, ^22^)^. To date, research on the physiological 24-h relationship between BP and IOP in treatment-naive, non-glaucoma patients with varying BP statuses is lacking. Our findings revealed a significant difference in the 24-h ocular volume between patients with and without nocturnal BP decline, regardless of their 24-h average BP level and hypertensive status.

No correlation was observed between BP and baseline IOP or the 24-h ocular volume. Considering the measurement error of 1 mmHg of Goldmann applanation tonometry and a relatively small difference in SBP (20 mmHg) between the normotensive and hypertensive patients, our pilot study was possibly underpowered for detecting the modest correlation of 0.2-0.3 mmHg per 10 mmHg of SBP observed in large clinical trials^(^6^)^.

Studies on the 24-h ocular volume have used various parameters for group comparisons, such as the 24-h ocular volume slope, AUC, time to large peak, number of large peaks (>90 mV), and variability of the mean^(^17^,^23^,^24^)^. However, evidence for choosing a single, uniform parameter for every analysis is insufficient, and comparing every previously defined parameter of the 24-h ocular volume curve reduces the study’s statistical reliability. We arbitrarily selected mean CLS output at night and during the day, the slope of the regression line, the variability of the mean, and several large peaks (>90 mV).

Previous 24-h studies investigating the relationship between BP and lOP have revealed no significant difference between day-time and night-time lOP in dippers and non-dippers, as well as between normotensive and hypertensive patients. However, nocturnal awake or seated measurements and the enrollment of patients on antiglaucoma or/and antihypertensive medications influenced these studies^(^25^, ^26^, ^27^)^. lnterestingly, while clinical studies have reported that a nocturnal drop accelerates glaucoma progression, experimental studies in non-human primates have demonstrated that BP alterations, in contrast to lOP changes, do not significantly affect optic nerve head blood flow^(^28^)^. Our findings that associate the dipping profile of BP with the increased 24-h ocular volume (a parameter linked to lOP) help explain previous conflicting observations regarding accelerated glaucoma progression in patients with nocturnal BP drop^(^27^)^. Notably, because sound scientific evidence on changes in blood flow in the optic nerve in response to nocturnal drop is lacking, we believe that increased nocturnal lOP in dippers, which has not been previously detected because of technical difficulties, might be associated with a higher risk of glaucoma progression in these patients.

The lack of difference in RNFL thickness between the dippers and non-dippers in our study is contradictory to the aforementioned findings. However, these results should be interpreted cautiously, as our patient group was relatively young (49 years vs. the average age of glaucoma diagnosis in most studies, 60-70 years) and did not include glaucoma patients^(^21^)^.

Furthermore, we hypothesized that differences in the 24-h ocular volume in the dippers and non-dippers reflect varying sympathetic activities in these groups. Notably, a study has reported that dippers have significantly lower sympathetic impulsation than non-dippers. These effects were most remarkable during the night when no voluntary actions affect sympathetic impulsation^(^29^)^.

The study findings might have very sound clinical implications. Namely, as BP measurements and hypertension evaluation are conducted in a low-cost primary care setting, identifying a direct link between the BP profile and ocular hypertension (or risk of glaucoma) might increase the cost-effectiveness of glaucoma screening.

The study limitations should be underlined. To fully comprehend the role of hypertension and nocturnal BP dip in glaucoma, a prospective study involving glaucoma patients, following a washout period, should be conducted. However, enrolling treatment-naive, hypertensive patients with glaucoma is difficult as hypertension is typically diagnosed at an earlier age, and withdrawal of antihypertensive medications is difficult to accept ethically. Furthermore, studies with larger sample sizes are required to confirm our findings.

In conclusion, night-time BP drop, but not the 24-h BP level, is associated with an increase in the 24-h ocular volume slope. Additional studies are warranted for evaluating whether accelerated glaucoma progression in dippers is primarily associated with low BP or high lOP, or whether a mixed mechanism is involved.
